# Albatross movement suggests sensitivity to infrasound cues at sea

**DOI:** 10.1073/pnas.2218679120

**Published:** 2023-10-09

**Authors:** Natasha Gillies, Lucía Martina Martín López, Olivier F. C. den Ouden, Jelle D. Assink, Mathieu Basille, Thomas A. Clay, Susana Clusella-Trullas, Rocío Joo, Henri Weimerskirch, Mario Zampolli, Jeffrey N. Zeyl, Samantha C. Patrick

**Affiliations:** ^a^School of Environmental Sciences, University of Liverpool, Liverpool L3 5DA, United Kingdom; ^b^Ipar Perspective Asociación Karabiondo Kalea, Bilbao 48600, Spain; ^c^Research and Development Seismology and Acoustics, Royal Netherlands Meteorological Institute, Utrecht 3731GA, Netherlands; ^d^Department of Geoscience and Engineering, Delft University of Technology, Delft 2628CD, Netherlands; ^e^Department of Wildlife Ecology and Conservation, Fort Lauderdale Research and Education Center, University of Florida, Davie, FL 33314; ^f^Institute of Marine Sciences, University of California, Santa Cruz, CA 95064; ^g^Department of Botany and Zoology, Stellenbosch University, Cape Town 7602, South Africa; ^h^Global Fishing Watch, Washington, DC 20036; ^i^Ecology of Marine Birds and Mammals, Centre d’Étude Biologique de Chizé, Villiers-en-Bois 79360, France; ^j^International Monitoring System Division, Comprehensive Nuclear-Test-Ban Treaty Organization, Vienna 1400, Austria

**Keywords:** Animal navigation, movement ecology, animal movement, seabirds, avian hearing

## Abstract

The ways in which seabirds navigate over very large spatial scales remain poorly understood. While olfactory and visual information can provide guidance over short distances, their range is often limited to 100s km, far below the navigational capacity of wide-ranging animals such as albatrosses. Infrasound is a form of low-frequency sound that propagates for 1,000s km in the atmosphere. In marine habitats, its association with storms and ocean surface waves could in effect make it a useful cue for anticipating environmental conditions that favor or hinder flight or be associated with profitable foraging patches. However, behavioral responses of wild birds to infrasound remain untested. Here, we explored whether wandering albatrosses, *Diomedea exulans*, respond to microbarom infrasound at sea. We used Global Positioning System tracks of 89 free-ranging albatrosses in combination with acoustic modeling to investigate whether albatrosses preferentially orientate toward areas of ‘loud’ microbarom infrasound on their foraging trips. We found that in addition to responding to winds encountered in situ, albatrosses moved toward source regions associated with higher sound pressure levels. These findings suggest that albatrosses may be responding to long-range infrasonic cues. As albatrosses depend on winds and waves for soaring flight, infrasonic cues may help albatrosses to identify environmental conditions that allow them to energetically optimize flight over long distances. Our results shed light on one of the great unresolved mysteries in nature, navigation in seemingly featureless ocean environments.

## Significance

Among animals, albatrosses are spectacularly mobile, yet the cues guiding long-distance movement across open ocean remain poorly understood. Of several candidate sensory mechanisms, including olfaction and magnetoreception, none provide sufficient explanation for the ability of albatrosses to find prey and anticipate atmospheric conditions optimal for energy-efficient flight. We investigated whether microbarom infrasound, sound below 20 Hz known informally as ‘the voice of the sea’, might be used as a movement cue by albatrosses. By comparing flight trajectories of individual birds to maps of modeled microbarom infrasound in the environment, we found that albatrosses preferentially move toward regions of ‘loud’ infrasound. This study provides an indication that free-ranging seabirds may use infrasound information to guide oceanic movement.

Predicting and responding to changes in environmental conditions is an essential part of animal movement. Over small spatial scales, such decisions may be guided by short-range cues such as vision and olfaction (1, 2), but it remains unclear what cues underlie movement at scales exceeding 100 to 1,000s km (3). In 1979, infrasound, inaudible low-frequency sound that propagates over thousands of kilometers in the atmosphere, was first proposed as a potential sensory cue following experiments in homing pigeons (4). Since then, observations of behavioral responses to infrasound anomalies from both environmental and anthropogenic sources have hinted at potential sensitivity of a much wider range of animals to infrasound (5–9). Its ubiquity in the environment (10), association with topographical- and weather-related features, and long-range propagation make infrasound a potentially useful cue that provides ecologically relevant information for movement, either as a long-range navigational map or by allowing animals to anticipate environmental conditions that favor or hinder movement (11, 12). Several mammalian and bird species have been identified as having hearing sensitivity in the infrasonic range (11) [e.g., Asian elephant *Elephas maximus* (13), black-tailed prairie dog *Cynomys ludovicianus* (14), chicken *Gallus gallus domesticus* (15), and domestic pigeon *Columba livia* (15)]. However, to date, no studies have directly modeled behavioral responses to in situ measures of this potential cue, and it thus remains unknown whether infrasound may be a missing part of the puzzle of how animals undertake very large-scale movements.

Compared to the terrestrial environment, where animals often orient using temporally stable landmarks, marine habitats are highly dynamic and typically lack visible features. Microbaroms (0.1 to 0.6 Hz) are one of the dominant infrasound sources within the marine environment and are radiated by standing ocean waves that are produced when counterpropagating waves of equal frequency meet and interfere constructively (*SI Appendix*, Fig. S1) (10, 16–20). Such conditions can prevail when surface waves driven by ocean storms interact with the background swell field. Infrasound can be detected over thousands of kilometers, potentially providing useful cues for large-scale oceanic movement (Movie S1) (12). Seabirds show some of the longest-distance movements in the animal kingdom and are highly responsive to changes in their environment. Among seabirds, wandering albatrosses (*Diomedea exulans*) exhibit spectacularly long-distance foraging trips, covering over 10,000 km in a single trip (21). During this time they are highly sensitive to changes in wind speed and direction, which strongly dictate their movement efficiency (22–24). Strong winds may improve flight conditions for albatrosses due to their high wing loading (23) or may increase prey availability or accessibility (22, 25, 26). However, excessively strong winds can also be maladaptive, leading to stranding, wing damage, or even death (7, 27). Indeed, recent evidence suggests that certain wind-adapted seabirds alter their behavior to align with wind patterns. For instance, streaked shearwaters (*Calonectris leucomelas*) track the eyes of storms, possibly to reduce the danger associated with powerful onshore winds (28). Additionally, some species, including wandering albatrosses, have been observed to avoid the strongest winds available to them (29). These findings together suggest that procellariform seabirds have a sensory mechanism that facilitates the detection of approaching storms (28). Such sensory cues might also allow albatrosses to anticipate and react to shifts in wind patterns over long distances, therefore aiding the construction of energetically efficient flight paths.

Albatrosses are highly adapted for soaring flight, traveling vast distances with minimal wing flapping. Two predominant flight modes allow albatrosses to extract energy from their physical environment. In dynamic soaring, albatrosses exploit vertical wind speed gradients to extract energy by moving from slower- to faster-moving air. In wave-slope soaring, albatrosses extract energy from the updrafts generated by wind blowing over waves (30). Through these methods, albatrosses can gain extra energy for soaring flight above that which can be extracted from wind conditions alone (30, 31). Areas with consistently strong winds generate swell and large waves, ideal for both wave-slope and dynamic soaring. As swell can persist in the absence of strong winds, there are likely evolutionary benefits to being able to detect regions with large waves, as they may provide birds with stable soaring conditions. Indeed, birds have been reported to follow waves for long distances even in conditions with little or no wind (30, 32). Microbarom source regions are louder in areas with colliding waves and may be stronger in stormy areas, with the intensity of this decreasing inversely with distance (10, 33). Consequently, microbaroms could provide a gradient field indicative of the strength and position of wavey and windy areas that are 100 to 1,000s km away (12, 28). The association between microbaroms and strong winds or stormy weather (34) could therefore make microbarom source regions a useful long-distance predictor of the presence of optimal waves and winds that minimize movement costs for wandering albatrosses (12, 35, 36).

The perception of sound by birds at microbarom frequencies (0.1 to 0.6 Hz) has been tested (and confirmed) in only one species, the homing pigeon (4). There are currently no empirical data on hearing abilities of albatrosses, but most birds hear best below 10 kHz with median best sensitivity at 2 kHz (range 0.99 to 4.03 kHz) and 9.6 dB (range −20 to 21 dB) (37). While six bird species are directly confirmed to perceive infrasound via audiometric tests, with sensitivity thresholds typically above 50 dB and below 20 Hz (38), most behavioral tests of infrasonic hearing sensitivity in birds proceed only down to a few Hz (11). This is likely due to the challenge of producing sounds <1 Hz in open, free-field conditions, which require large sound sources and high amplitudes (11). Therefore, examining behavioral responses of wild animals in free-ranging conditions could help fill these critical gaps in our knowledge.

To examine the behavioral responses of wandering albatrosses to infrasound, we investigated whether individual movement decisions were influenced by the microbarom soundscape during foraging trips. We developed microbarom soundscape maps, allowing us to model microbarom sound pressure from the perspective of individual birds at any given location and time (20). Soundscapes methods were validated using in situ infrasound measurements recorded by bespoke INFRA-EAR biologgers attached to birds in addition to 5 y of continuous infrasound array data measured as Kerguelen Island (39). Using high-resolution GPS tracks collected from 89 individuals breeding on the sub-Antarctic island of Crozet, in the Southern Ocean, we constructed habitat selection models to test whether individuals showed directional preference according to the sound pressure they experienced while in flight, after controlling for contemporaneous wind conditions experienced by birds. This study uses microbarom soundscapes to quantitatively explore behavioral responses to infrasound in a free-ranging bird.

## Results

**Fig. 1.**
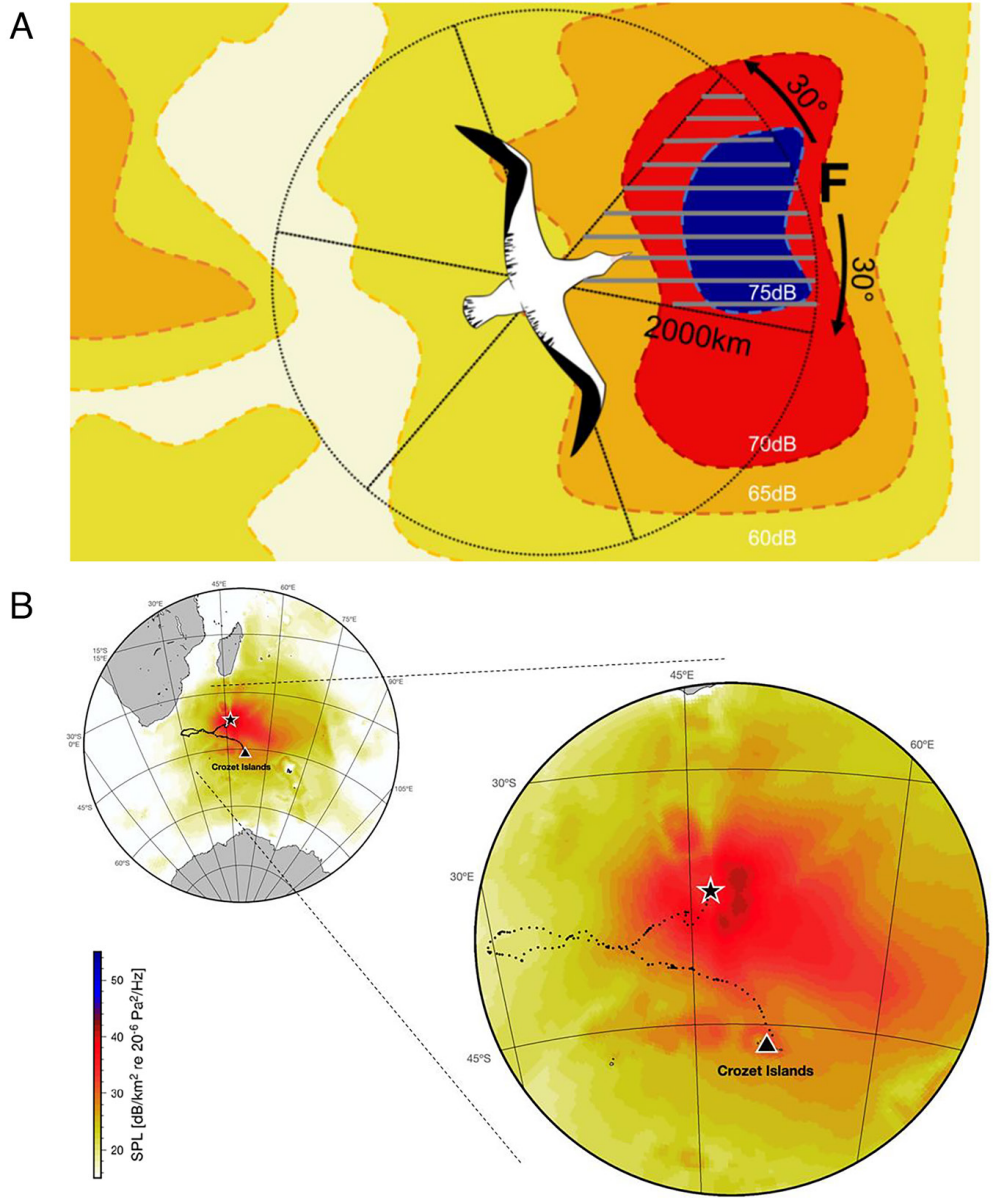
(*A*) Schematic to illustrate the analysis approach. Analysis was performed on sections of GPS tracks identified as flight and lasting for at least 20 km. The bird’s bearing at the decision point was calculated, and the area 30° either side of this bearing used to define a 60° ‘focal’ segment (labeled ‘F’; striped) emanating from the bird’s position. Numerically summed sound pressure across a 2,000 km radius in this focal segment was compared to the remaining five unchosen but available segments around the bird. The microbarom soundscape is represented by colored contours indicating sound pressure level (SPL) at different decibels to show that the ‘focal’ segment contains higher dB SPL. (*B*) Plot of a static microbarom infrasonic soundscape (0.06 to 1.1 Hz) at 00:00 UTC (2013-02-09) from the perspective of a foraging albatross (i.e., along the bird’s trajectory) with zoomed map. SPL (indicated by the color scale) represents the summed SPL that the bird can perceive, accounting for propagation paths and losses. The bird’s position is indicated with a black star and the beginning of the track with a black triangle (from the colony on Crozet Island). See Movie S1 and *SI Appendix*, *Supplementary Files*, for a dynamic map of the infrasound landscape. In both figures, SPL is used for illustration for interpretability; analyses were conducted using sound pressure measured in pascals.

We analyzed the directional preference of wandering albatrosses in relation to available microbarom sound pressure as well as wind speed and direction. This analysis depended on identifying ‘decision points’, points at which the bird decides upon a direction to head in. After splitting GPS tracks into bouts of either directed flight, searching, or resting behavior, we isolated bouts of directed flight exceeding 20 km and extracted the first GPS fix in each bout as the ‘decision point’ (Fig. 1*A*). The area surrounding the bird from each decision point was split into six segments of equal size (60°), and the environmental conditions in each segment were compared to the segment that contained the bird’s actual flight path (the ‘focal’ segment; Fig. 1*A*).

We analyzed 3,175 individual decision points (1,852 for females and 1,323 for males), with a mean of 35 per trip. As previously reported (22), males exhibited a more southerly foraging distribution than females (*SI Appendix*, Fig. S2). As our models compared conditions within the decision points, we could not fit the variable of ‘sex’ in our models, and so we modeled males and females separately to account for any sex-related differences in responses to infrasound.

**Fig. 2.**
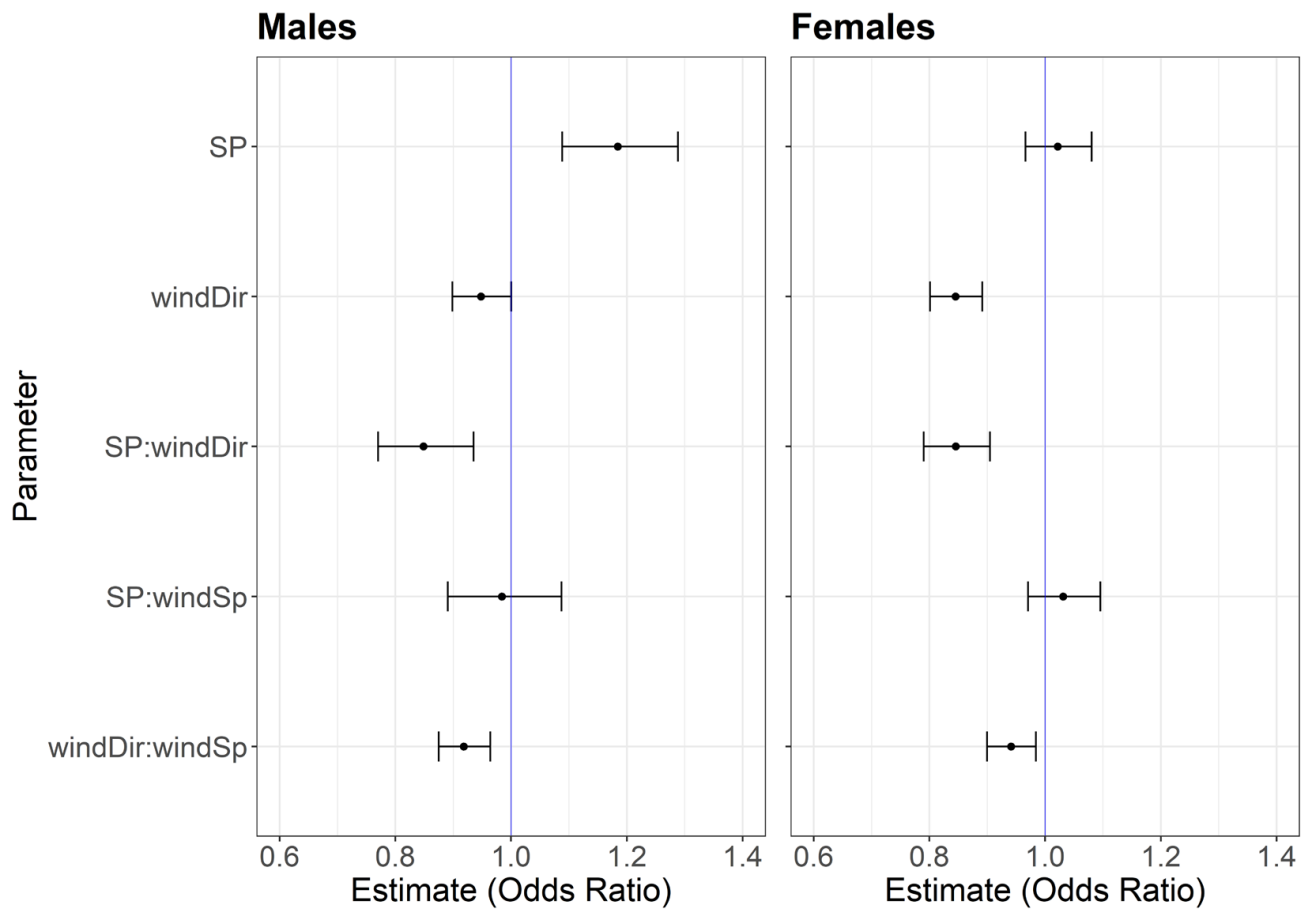
Effect of sound pressure, wind direction, and wind speed on male and female albatross movement decisions. Coefficient estimates represent the effect of each parameter and their interactions on the probability of segment selection from the best-supported model for each sex (males = wind+SP model; females = wind + SP model), given as mean ± SE. SP = microbarom infrasound pressure, pascals; windDir = wind direction relative to segment bearing, °; windSp = wind speed, m/s. Parameters joined by ‘:’ indicates interactive effect. As wind speed does not vary within decision points, it could not be fitted as a fixed effect (*SI Appendix*, *Supplementary Materials*). Coefficient estimates are given as odds ratios, which quantify the strength of an association between each parameter and its outcome (in this case, selection of focal cone). Coefficients whose CI do not overlap 1 (blue line) are deemed to have significant influences on movement decisions, with coefficients >1 indicating a positive response and coefficients <1 indicating a negative response. Both males and females preferentially selected segments with tailwinds and high infrasound.

We tested whether albatrosses preferentially moved toward areas of higher sound pressure during their foraging trips, by comparing sound pressure values in focal segments to the five remaining segments in the decision point (Fig. 1*A*). We fitted two competing models to segment preference, one containing wind variables alone and another containing wind variables and sound pressure. We assessed the relative support for each using QIC (Quasi-likelihood under independence criterion), likelihood ratio tests, and concordance indices (Table 1) and found the strongest support for the wind+SP model (Table 1 and Fig. 2) in both sexes.

**Table 1.** Comparison of Quasi-likelihood under Independence Criterion (QIC) parameters for model selection between the Wind model and the Wind+SP model

Model name	Variables	*K*	QIC	QIC*ω*	Χ^2^	C-index
*Females*						
Wind+SP	SP * windDir + SP:windSp + windDir:windSp	5	6336.97	0.99	92.92	0.61
Wind	windDir * windSp	2	6360.42	< 0.001	62.12	0.56
*Males*						
Wind+SP	SP * windDir + SP:windSp + windDir:windSp	5	4729.39	0.99	71.94	0.61
Wind	windDir * windSp	2	4770.18	< 0.001	17.69	0.53

K = number of parameters; QICω = QIC weight, assesses weight of evidence in support of model; Χ^2^ = compares log-likelihood of the full model to the log-likelihood of a null model; C-index = concordance index, measures agreement between model predictions and observed response. Smaller QIC and higher QICω indicate better support for the model; larger Χ^2^ values indicate a better model fit. Parameters joined by ‘:’ indicate an interaction; parameters joined by ‘*’ indicate an interaction plus the main effects of the singular variables. As wind speed does not vary within decision points, it could not be fitted as a fixed effect (*SI Appendix*, *Supplementary Materials*). The model name indicates how the model is referred in the text. Models are ordered by QICω. Notes – SP = sound pressure, pascals; windDir = wind direction relative to segment bearing, °; windSp = wind speed, m/s.

**Fig. 3.**
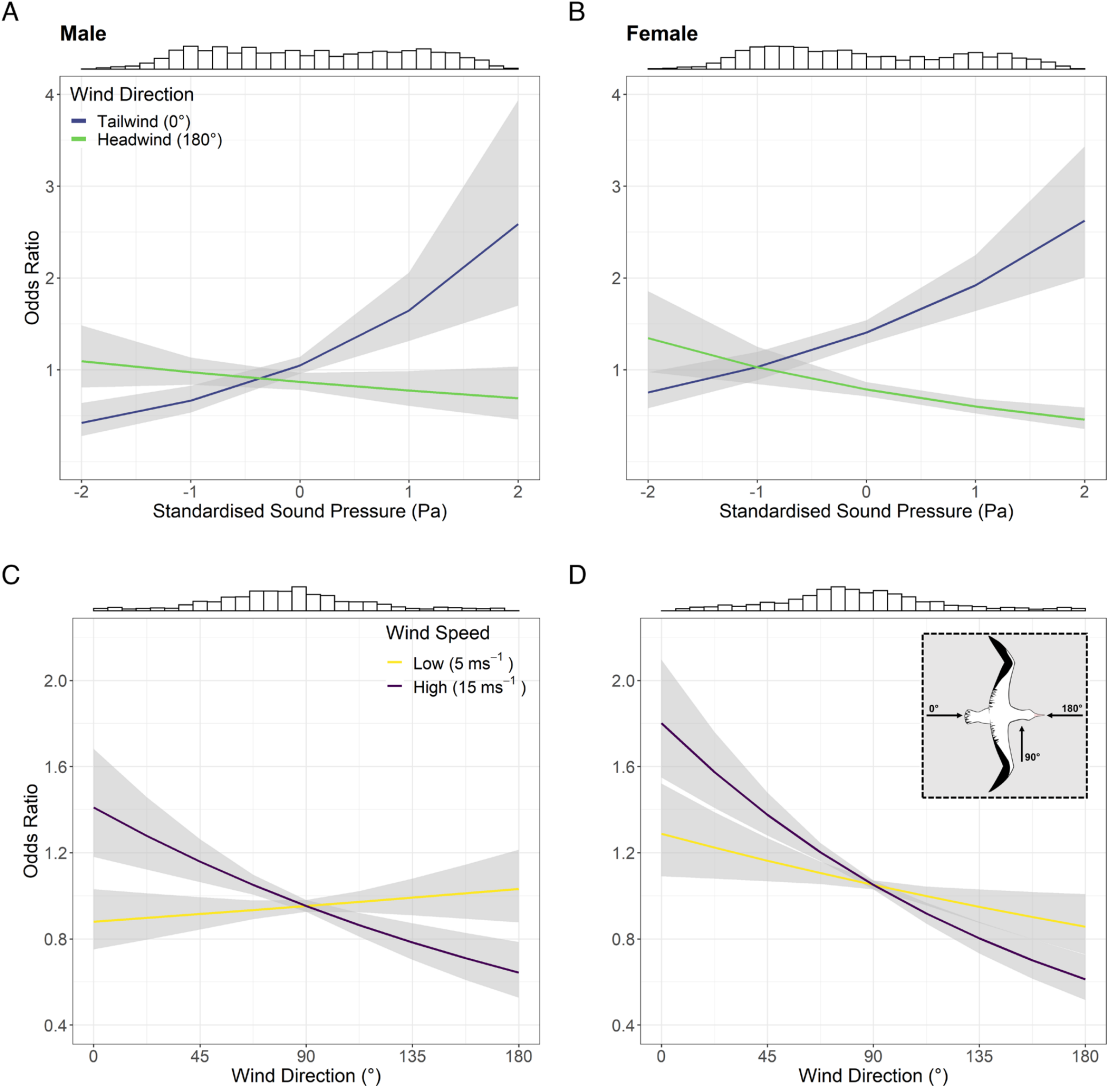
Effects of (*A* and *B*) sound pressure level, Pa and relative wind direction, ° (where blue = tailwind and green = headwind) and (*C* and *D*) relative wind direction and wind speed (where yellow = weak winds, purple = strong winds) on segment selection for males (*Left*) and females (*Right*). Male and female models were fitted separately and so are presented in separate panels. The cartoon *Inset* in (*D*) indicates wind direction relative to direction of travel of bird. Histograms on top of plots indicate distribution of SP (*A* and *B*) and relative wind direction (*C* and *D*) in focal cones. Gray shaded areas indicate 95% CI of prediction. Odds ratios quantify the strength between two variables and in this case indicate the likelihood of selecting a segment containing a given value for each variable.

In tailwinds, both males and females were more likely to choose segments with higher sound pressure, although there was limited effect of sound pressure in headwinds (Table 1 and Fig. 3 *A* and *B*). In strong winds, both males and females were more likely to select segments in tailwinds; in weak winds, males showed a slight preference for headwinds, while females continued to exhibit preference for tailwinds (Fig. 3 *C* and *D*).

## Discussion

Where previous evidence for infrasound responsiveness in birds has been largely incidental (6, 7, 40), our infrasound soundscapes allowed us to directly model albatross movements in relation to the microbarom sound pressure levels they experienced. Given the subset of models we compared, we found stronger support for the model containing sound pressure, providing correlative evidence that movement decisions in wandering albatrosses are consistent with behavioral responsiveness to microbarom infrasound. On commencement of periods of sustained flight, birds were more likely to move toward regions where sound pressure was high. Albatrosses also responded to instantaneous wind conditions, preferentially traveling with strong tailwinds, which is concordant with previous findings (22). Even accounting for these responses to wind, we also observed a preference for high sound pressure regions, suggesting that albatrosses may be responding to long-range propagation of microbarom infrasound in addition to wind conditions they were experiencing in situ, although future work is required to determine the spatial scale over which this response operates. In other words, albatrosses may use favorable winds to orientate toward areas containing high sound pressure. Overall, our results provide an indication that infrasound may be an important, previously unidentified cue for seabird movement.

Albatrosses are highly dependent on wind and wave energy for dynamic and slope soaring (30, 41) and are generally restricted to oceans where wind speeds are consistently high (42). However, albatrosses also inhabit subtropical oceans where wind speeds are often low and in the absence of strong winds are thought to strongly depend on ocean waves for slope soaring (31, 41). The high wing loading of wandering albatrosses results in high energetic costs associated with flapping flight (30, 31, 43, 44). Consequently, albatrosses seek to take advantage of waves and winds to soar whenever possible. While instantaneous wind conditions are therefore clearly important, the ability to predict wave and wind conditions at much further distances may be an important factor shaping their movement decisions. Although spatial memory may allow wandering albatrosses to relocate profitable foraging areas, their tendency to take long, looping trajectories to opportunistically feed on widely dispersed prey means they are unlikely to visit the same sites repeatedly, reducing the value of such memory (45). As a result, wandering albatrosses may particularly benefit from predicting changes in environmental conditions over long ranges, and potentially in areas they have not recently visited. As seen in albatross GPS tracks (see, for instance, Movie S1), albatrosses do not seem to visit distinct patches in the ocean—rather, their movement seems to match the dynamic landscape created by the microbarom field at a large scale. Microbarom infrasound occurs where there are counterpropagating ocean waves, which may, for example, be swell- or storm-induced (46). As a potential indicator of strong winds and high waves beyond their visual range, microbarom infrasound may therefore provide an important cue that allows albatrosses to commute toward locations that allow them to search for prey while minimizing movement costs.

While we found evidence that both male and female albatrosses responded to sound pressure in their movement decisions, these effects were stronger for males. This probably reflects sex-specific differences in the benefits of pursuing particular sea conditions. Males are 20% larger in both mass and wingspan than females and are therefore likely more dependent on winds than females during their foraging trips (22, 43, 47). Due to their lower wing loading, females may be under less pressure to seek out particularly windy or wavy foraging areas (43, 48) or may avoid extreme weather conditions that may lead to structural wing damage (27). These differences may also reflect the foraging destinations of males versus females breeding at Crozet. While males are known to forage in turbulent southerly locations where they routinely fly through low-pressure systems, females tend to forage in northerly locations with more persistent high-pressure systems, which have calmer seas and therefore lower sound pressure (*SI Appendix*, Fig. S2 and ref. 22).

The reliance of wandering albatrosses on a soaring flight style that takes advantage of vertical wave and wind gradients means that any ability of birds to use cues other than the winds they experience, and to anticipate and respond to changes in soaring conditions, will be extremely advantageous. Microbarom infrasound is pervasive and long ranging, meaning that it is a likely useful source of information at large scales extending beyond the ranges of other sensory modalities such as vision and olfaction (12). However, further research is needed to determine its exact relationship with weather conditions, and the precise distance over which albatrosses may be responding to it. Furthermore, it remains possible that birds are responding to other environmental cues, such as barometric pressure. In combination with data on other potential environmental variables, future analyses making use of meteorological and geophysical models may help to determine the precise role of microbarom infrasound in movement decisions, as well as tease apart the relative contribution of other putative cues, with microbarom infrasound likely to form part of a multimodal sensory system (12).

Having garnered correlative evidence that albatrosses may be behaviorally responding to infrasound, a next step is to establish the mechanisms by which albatrosses detect direction or heading from it. Currently, knowledge about avian directional hearing mechanisms at infrasonic frequencies is limited (reviewed by refs. 11 and 12). The lowest limit frequency threshold found in pigeons (~0.1 to 1 Hz) was determined with a sound source of >100dB (11), which is below the dB levels typically associated with oceanic ambient noise. Frequency thresholds remain unknown for wandering albatrosses, but given knowledge about the inner ear systems in albatrosses should encapsulate very low frequencies, with predicted frequencies lower than that for pigeons (*SI Appendix*). Birds could theoretically detect small infrasonic frequency shifts (12, 49), which could be used to determine sound direction by exploiting the Doppler shift (12), but sound localization performance within noisy pressure field conditions is an unknown added complexity. Lab-based testing of hearing sensitivity may provide complementary information, but presents significant technical challenges, especially when investigating perception of very low-frequency sound, such as microbaroms (11, 12). Within birds, albatrosses are good candidates for low-frequency sound perception given their large size. In many animal groups, bigger ears are associated with shifts in the hearing response to lower frequencies (50, 51). In birds, larger body size is associated with lower frequencies of best sensitivity (52), and a recent study indicates that larger ear structures in birds are related to improved sensitivity and extended low and high hearing limits (37). Given the differences in behavioral response between males and females, it would also be useful to investigate sex differences in auditory processing and sensitivity to infrasound, which are well documented in animal groups at higher, noninfrasonic frequencies (53–55).

Here, we have used a wild, free-ranging bird to provide correlative evidence that infrasound may play a role in shaping individual movement decisions in a foraging context. While in wandering albatrosses, microbarom infrasound might be most useful as an indicator of advantageous wind conditions and sea states, the effect of bathymetry such as continental shelf edges on microbarom sound pressure might also make infrasound a useful cue for the detection of neritic areas for homing and navigation in other species (10, 18, 33, 35). At present, the challenges of experimentally testing hearing capabilities of animals have restricted its study to a very small number of species. Consequently, studying free-ranging movement may make a valuable contribution to understanding the sensory perspectives of animals. The development of methods to collect real-time data on infrasound from the perspective of equipped animals (39) in combination with our infrasound soundscape maps will promote investigation into the effect of infrasound on the behavior of birds and other taxa, giving further insight into the nature of long-range movement in the relatively featureless environment of the ocean.

## Materials and Methods

### Bird Data Collection and Processing.

Data were collected from wandering albatrosses breeding on Crozet Island (46°24′S; 51°46′E) between 21st January and 31st March 2013, corresponding to the incubation period. A total of 89 birds (50 females and 39 males) were individually sexed, marked, and fitted with GPS loggers (IgotU 120/600 Mobile Action Technology^©^). Loggers were programmed to obtain a fix every 15 min and were dorsally attached using thin strips of marine Tesa^©^ tape. Instrumented birds were recaptured after they had completed at least one foraging trip, and the longest foraging trip for each bird was retained for analysis.

All data processing and analyses were conducted in R version 4.1.1 (56). We identified foraging trips by defining the start of trips as the first departure of at least 10 km from the colony, and the end as the first fix following return to the colony. All GPS fixes taken less than 10 km from the Crozet shelf at the beginning and the end of each trip were removed from the analysis, as fishing vessels, to which wandering albatrosses are attracted, operate in this area and this attraction could dilute the effects of environmental variables on movement decisions (22). We used hidden Markov models (HMMs) to identify three behaviors—directed flight, searching, and resting on the water. We used the momentuHMM R package (57) to fit three-state generalized HMMs to the GPS tracks and following methods by ref. 58. HMMs were fitted using the input variables of step length and turning angle and categorized fixes into discrete behavioral states as follows: directed flight (small turning angles and high speeds), searching (moderate to wide turning angles and moderate speeds), and rest (small to moderate turning angles and low speeds).

We followed previous methods (22) to compare expertly assigned behaviors with the output of the HMM used to assign behavioral states to ensure that model classification was biologically appropriate. Briefly, three trips were randomly selected for expert assessment, representing 3,133 fixes. An observer, who was blind to the output of the HMM, manually inspected the GPS trajectories and classified behavioral states (search, travel, and rest) based on movement patterns. Model accuracy was determined as the percentage of fixes where both the model output and the expert classification agreed. Overall accuracy was high at 81.1% and matching findings in other studies (22, 59), with travel, our behavior of interest, showing very high accuracy at 93.5%.

### Environmental Data and Processing.

Hourly wind data were obtained at a 0.28° spatial resolution, corresponding to 15 to 30 km, from the European Centre for Medium Range Weather Forecasts (ECMWF) ERA5 reanalysis dataset [Copernicus Climate Change Service (CS3), 2017]. We took data from 10 m above sea level, which is as close as possible to the average observed height (8 m) for wandering albatrosses (41).

Microbarom (0.06 to 1 Hz) soundscape maps were constructed for the purposes of this study using previously developed ECMWF model data (20); the methodology is described in detail (39) and produced reconstructed soundscapes within 2.7 dB for 85% of measurements in the microbarom band. The soundscape maps were validated by comparing the modeled soundscape with infrasound data recorded simultaneously using the INFRA-EAR biologger, deployed on wandering albatrosses in 2020 (36) as well as an infrasound array IS23 (Kerguelen) that is part of the International Monitoring System for the verification of the Comprehensive Nuclear-Test-Ban Treaty (35, 36).

Microbarom soundscapes can be reconstructed based on evanescent and propagating microbarom components. Evanescent microbaroms at the ocean–air interface are a direct product of traveling ocean surface waves, disregarding water depth or bathymetry, and decay vertically (46, 60). The nonlinear interaction of countertraveling ocean surface waves results in standing ocean waves, causing the radiation of acoustic energy and resonance within the water column (61, 62). At the interface of the water column, acoustic energy is radiated into the atmosphere as propagating microbaroms (16). Evanescent microbaroms are only detectable right above the source area and peak around 0.1 Hz (36), while propagating microbaroms can travel over large distances. The received microbarom infrasound level depends on the source level, with higher source levels giving larger propagation distances. Considering typical microbarom amplitude levels and maximum distances to microbarom source regions, a typical microbarom propagation distance would be over 5,000 km, with a radius of 2,000 km capturing 95% of the acoustic signal (hence our decision to use this radius in our analyses), but may be carried much further, up to 10000km. (up to ~10,000 km) and typically peak around 0.2 Hz (17) (see ref. 20 and *SI Appendix* for a detailed explanation). Since the research question within this study focused on whether seabirds use infrasound as a cue for long-range movement, only the propagating microbarom components, which are stable, were of interest. Therefore, the soundscapes were reconstructed based on only the propagating microbarom component.

The soundscapes were reconstructed using ERA5 atmospheric and wave reanalysis models to 1) calculate the initial sound pressure level produced by each position of the ocean (10, 18) and 2) calculate the propagation and absorption of the sound signals within the atmosphere between the source areas and the bird’s position (63, 64). A stereographic polar grid was calculated from the perspective of the bird’s position, for each GPS location and time at the start of a decision point. Over this grid, the initial source model (18) and the propagation model (64) were interpolated. Further detail on soundscape construction and propagation of microbarom infrasound can be found in *SI Appendix*.

### Defining Decision Points.

To test whether wandering albatrosses are responsive to microbarom infrasound in their movement decisions, we compared the estimated sound pressure (SP), measured in pascals, in the observed heading direction of individual birds with what was available around the bird at specific points within each trip, where we hypothesized that the bird decides on a direction to move in. We named these points ‘decision points’.

Decision points were defined as the first GPS fix within a bout of directed flight lasting for at least 20km following a period of searching or resting, as this represents a period of long-range travel, where we predict microbarom infrasound to be most informative. At each decision point, a circle with a radius of 2,000 km emanating from the bird’s position was divided into six segments with a 60° aperture (hereafter ‘segment’; Fig. 1*A*). We conducted a post hoc sensitivity analysis to determine whether changing the aperture size led to variation in our results and found no effect (*SI Appendix*, Fig. S3). The segment containing the true bearing of each albatross, known as the ‘focal’ segment, was placed ±30° from the exact bearing of the bird at each decision point (Fig. 1*A*). As travel is defined within the HMM partly by its shallow turning angles (range: −0.55 to 0.58 radians), the heading of the bird at this point is likely to reflect the trajectory over the rest of the travel bout.

For each decision point, the closest microbarom propagation map in time (within 1 h) was associated with the GPS fix (36). Total SP was numerically integrated over the area of each segment and then *z*-standardized within a decision point to account for temporal changes in SP levels. A radius of 2,000 km was selected as it has been estimated that microbarom sources up to 2,000 km from the bird’s position contribute 95% of the total acoustic power (20); in other words, beyond 2,000 km microbarom infrasound levels would make negligible difference to the bird’s perceived SP. This was a conservative decision, as our intention was to examine whether albatrosses could detect and behaviorally respond to infrasound at all. As microbarom maps were produced for sea areas and not land, some segments had a radius of less than 2,000 km due to the land overlap. To promote comparability across all segments, we removed from the analysis one decision point that, due to this land overlap, contained segments with radii of less than 2,000 km.

### Statistical Analysis.

We tested whether albatrosses preferentially moved toward areas of higher microbarom infrasound during their foraging trips, by comparing SP levels in focal segments to the five paired segments that were not selected. To this end, we fitted conditional logistic regression models using the R package survival (65), which explicitly account for the nonindependence of segments within each decision point, which is not possible using classical habitat selection models. Using this method, we could compare conditions in chosen direction (‘focal’ segments) versus alternative options (Fig. 1*A*). Each decision point was clustered within the variable ‘bird identity’ to control for repeated measures from the same individual. All continuous variables were scaled to facilitate comparison.

Wandering albatrosses are known to respond to instantaneous wind direction and speed in their movement decisions (22). We therefore aimed to test whether including SP in our models better explained the segment preference of birds than instantaneous wind conditions alone. We first constructed a baseline model that fitted segment preference as a function of wind conditions only (relative wind direction and its interaction with speed). Relative wind direction was calculated as the angular difference in direction between the center of the segment angle and wind direction, standardized to between 0° and 180°. We compared this against a competing model (Table 1) in which we fitted the interaction of wind direction and wind speed respectively with SP. The distinction of trip stages is not included in our models, as albatrosses undertake long looping trips that do not have distinct outbound, middle, and inbound sections (*SI Appendix*, Fig. S4).

As the structure of conditional logistic models means they explicitly compare segments within decision points, they can only be used to compare conditions that vary among segments within each decision point. We therefore were unable to fit sex or instantaneous wind speed as fixed effects, as these parameters do not vary within decision points. To account for the effect of these variables on segment preference, we took two approaches. For wind speed, we fitted the variables as interactions with wind direction and SP, respectively. To assess whether the selection preferences of males and females differed, we fitted models to datasets containing males and females separately and compared their outputs.

We used an information-theoretic framework to compare and rank each of the three models using the quasi-likelihood under independence model criterion (QIC) and QICω (QIC weight, giving weight of evidence in support of model). We assessed model support using the QIC, where the lowest QIC indicates the best-supported model, and QIC weights, which assess the weight of support for each model (ω; specifically, the weight of evidence of model *i* being the Kullback–Leibler best model; (66, 67)). We additionally conducted a likelihood ratio test to compare the log-likelihood of the full model to its null, and calculated concordance indices to measure the agreement between the observed responses and predictors for all models.

## Supplementary Material

Appendix 01 (PDF)

Movie S1. **Animation to show hourly variation in the microbarom infrasonic soundscape (0.06-1.1 Hz) from the perspective of a foraging albatross.** Animation begins 2013-02-01 15:00 UTC and runs to 2013-02-16 10:00 UTC. Leftmost panel shows the initial hourly infrasound microbarom source model integrated between 0.1 and 1Hz, according to Waxler et al., 2007 and implemented by Smets, 2018. Middle panel shows the infrasound propagation loss model by Taillipied et al., 2017, from the perspective of the bird’s GPS location, again integrated between 0.1 and 1Hz. Arrows superimposed on the propagation loss model indicate the wind direction and speed within the stratosphere and troposphere. Rightmost panel shows the soundscapes from the bird’s perspective, between 0.1 and 1 Hz. The star indicates the GPS position of the bird at the presented timestamp. The dots show the previous positions, showing the track. The triangle indicates Kerguelen islands, where IMS infrasound station I23FR is stationed for the CTBTO. SPL is used for illustration for interpretability, analyses were conducted using sound pressure measured as pascals.

## Data Availability

All data and code used in the analysis are hosted on Zenodo at https://doi.org/10.5281/zenodo.6979710 (68).
