# Supplementary material for: Albatross movement suggests sensitivity to infrasound cues at sea
Source: Proc Natl Acad Sci U S A. 2023 Oct 9;120(42):e2218679120. doi: 10.1073/pnas.2218679120 (PMC10589618; doi:10.1073/pnas.2218679120)
Supplement: Supplementary file 1 — Appendix 01 (PDF) [file pnas.2218679120.sapp.pdf]

PNAS

Supporting Information for

Albatross movement suggests sensitivity to infrasound cues at sea

Natasha Gillies[1†], Lucía Martina Martín López[1,2†], Olivier F. C. den Ouden[3,4], Jelle D. Assink[3], Mathieu Basille[5], Thomas A. Clay[1,6], Susana Clusella-Trullas[7], Rocío Joo[8], Henri Weimerskirch[9], Mario Zampolli[10], Jeffrey N. Zeyl[7], Samantha C. Patrick[1*].

[1]School of Environmental Sciences, University of Liverpool; Liverpool, UK.
[2]Ipar Perspective Asociación, Karabiondo Kalea; Sopela, Spain.
[3]R&D Seismology and Acoustics, Royal Netherlands Meteorological Institute (KNMI); De Bilt, Netherlands
[4]Department of Geoscience and Engineering, Delft University of Technology; Delft, Netherlands.
[5]Department of Wildlife Ecology and Conservation, Fort Lauderdale Research and Education Center, University of Florida; Davie, USA.
[6]Institute of Marine Sciences, University of California; Santa Cruz, USA.
[7]Department of Botany and Zoology, Stellenbosch University; Stellenbosch, South Africa.
[8] Global Fishing Watch; Washington, US.
[9]Centre d'Étude Biologique de Chizé; Villiers-en-Bois, France.
[10]Comprehensive Nuclear-Test-Ban Treaty Organization (CTBTO); Vienna, Austria.

*Corresponding author: Samantha C. Patrick

Email: samantha.patrick@liverpool.ac.uk

†These authors have contributed equally to this work.

This PDF file includes:

Supporting text
Figures S1 to S5
Table S1 and S2
Movie S1

Supporting Information Text

Propagation and attenuation of infrasound

The received microbarom infrasound level at a receiver location depends on the source level and the propagation loss from microbarom source region to receiver. This implies that louder microbarom source regions can, under similar propagation conditions, propagate over larger distances than quieter microbarom regions. Similarly, equally loud microbarom regions that are located at a similar distance, but opposite directions from a receiver will not be detected with a similar signal level, because of different long-range propagation conditions.

The received microbarom signal can consist of multiple contributions from multiple microbarom source regions. Typically, the strongest contribution will mask the weaker microbarom signals. The distance to microbarom source regions greatly influences how many microbarom regions contribute to the recorded microbarom signal. Earlier studies (*1*, *2*) have shown that the microbarom signal on island stations consists of contributions from multiple directions in contrast to stations on the continent. Note that this finding is consistent with the microbarom soundscape analysis presented in this study.

From earlier microbarom soundscape analyses carried out for receivers at/near sea (*1*, *2*), it followed that a region of 2000 km captures 95% of the microbarom signal recorded on a remote infrasound station. Moreover, it was found that the most dominant microbarom contribution to the soundscape was located ~250 km away. While microbarom propagation distances over 5000 km are be possible for receiver locations that are located inland, it could be argued that such conditions are not relevant for the topic of interest.

The received signal level (S) from a certain source depends on the transmission loss (TL) that a signal, which is originally at a given source level (SL), experiences propagating from source to receiver. This can be summarized by the passive sonar equation (e.g., (*3*)):

$$\mathrm{S} = \mathrm{SL} - \mathrm{TL}$$

All quantities in the upper equation are expressed in decibels relative to some reference distance; typically 1 m is used. In this study, we hypothesize that the wandering albatross can detect infrasound and represents the receiver which detects signal level S.

The acoustic transmission loss is the combined loss due to geometrical spreading of sound and intrinsic absorption. Geometrical spreading describes the loss of energy as a wave propagates away from the source because the wave energy is spread out of an increasingly larger area. Intrinsic absorption describes the loss of wave energy as it propagates through air and is determined by the chemical composition and physical conditions of air (i.e., temperature and pressure) throughout the atmosphere (e.g., (*4*)).

The absorption of sound in air is proportional to the sound frequency squared. For this reason, sound waves at infrasonic frequencies experience relatively low loss rates compared to sound waves at higher frequencies. The standard model that describes the attenuation of sound throughout the atmosphere is the model published by (*5*), which is also used in this study. In the lower and middle atmosphere, losses are predominantly caused by vibrational relaxation for which acoustic energy is transferred to the vibrational modes of the air molecules (N_2, O_2, N, O, CO_2, O_3, and H_2O). Since the air becomes drier with increasing altitude, the attenuative effects of humidity (e.g., in the case of mist) are strongest in the lower troposphere. At upper atmospheric altitudes (e.g., in the mesosphere and lower thermosphere), acoustic energy is transferred into heat, due to so-called thermo-viscous absorption.

The acoustic transmission loss can be estimated by computing the loss along the propagation path through the atmosphere, which in turn is determined by the temperature and wind conditions

throughout the atmosphere. To a good approximation, these factors can be combined in a so-called 'effective sound speed'. Because the effective sound speed varies along the infrasonic propagation path, infrasound waves do not propagate along straight lines (as light does, approximately). Instead, infrasonic wavefronts bend upward (downward) for negative (positive) gradients in effective sound speed. Thus, infrasonic waves bounce and up and down throughout the atmosphere from source to receiver in an 'acoustic waveguide'. A large variety of sound propagation models exist that can be used to estimate the transmission loss (e.g (*6*, *7*)). In this study, we make use of the empirical propagation model published by (*8*).

The bottom of the waveguide is the Earth's surface. The top of the waveguide is an upper atmospheric layer, for which the altitude depends on several factors, including the effective sound speed conditions and the infrasonic propagation angles (e.g. (*6*)). The most important waveguide to consider for long-range propagation is the stratospheric waveguide (e.g., (*9*)), for which the top is near the stratopause around 50 km altitude. At this altitude, there is a large-scale wind-jet that is referred to as the circumpolar vortex or stratospheric jet.

Infrasound propagation is largely anisotropic because of the influence of the horizontal wind on the effective sound speed, and therefore the formation of acoustic waveguides. Infrasonic waves are more efficiently trapped in a waveguide for propagation directions that are like the direction of the wind jet. The circumpolar vortex reverses direction twice a year, during the equinox periods.

During the period of interest for this study (February), the circumpolar vortex is westward (see (*9*) for a climatological study). Therefore, it is to be expected that sources that are east ('upwind') of the receiver are more likely to be detected because of relatively low acoustic transmission loss. This also explains why the soundscape shown in Figure 1 (main manuscript) has an elongated shape extending towards the east of the wandering albatross.

Synthesis of microbarom soundscape maps

In this study, we make use of microbarom soundscape maps. These maps spatially quantify to which microbarom source regions a bird at any given position in space and time is sensitive to. The maps are essentially reconstructed by evaluating the passive sonar equation (previous section) for all possible source-receiver combinations over the grid.

A snapshot of a soundscape valid for 2013-02-09 00:00 UTC is shown in Figure 1 of the main manuscript. An animation showing hourly changes in microbarom infrasound from the perspective of a bird, captured between 2013-02-01 15:00 UTC to 2013-02-16 10:00 UTC is included in the supplementary files (Movie S1).

The soundscapes are discussed in detail in Section 3 of (*1*) and detailed examples are provided in studies by (*1*, *2*).

Here, a brief step-by-step summary of how the soundscapes are computed is provided.

1. Span a stereographic polar grid, from the perspective of the bird's position, for each GPS location and time at the start of a decision point. This is visualized in Figure 2a of (*1*).
2. Compute the microbarom source level maps using the microbaroms source model by (*10*) and model data from the ERA5 HRES 2DFD reanalysis ocean-wave model. Re-interpolate the resulting map onto the stereographic grid created at step 1. This is visualised in Figure 2c of (*1*).
3. Compute the transmission loss for propagation for each grid cell to the position of the bird using the propagation model by (*8*) and the atmospheric re-analysis model ERA5. Re-interpolate the resulting map onto the stereographic grid created step 1. This is visualised in Figure 2d of (*1*).
4. Multiply the results from steps 2 and 3 for each grid cell in the stereographic grid. To account for large propagation times between source region and receiver, each grid cell is

evaluated at the model is nearest to the observation time minus the propagation time. This means that simulated soundscape consists of microbarom source regions that exist throughout different hours of the day. This is visualised in Figure 2b of (*1*).

5. Evaluate the surface integrals over the areas that enclose microbarom sources to obtain the total acoustic power. This is visualised in Figure 4 of (*1*).

Audiogram predictions for wandering albatrosses

Using the model presented in (*12*), we predicted the frequency of the lower half of an audiogram at a level of 60 dB for wandering albatrosses. Based on columella volume, we used the known values for albatrosses to determine when their audiogram would cross 60 dB. Using the effect sizes presented in Zeyl et al. (2023) (Table S1), and a columella volume for wandering albatrosses of 1.45, we obtained a predicted lower frequency of 5.3 Hz at 60 dB (Y = 1.934 – 0.714X). This value is lower than the predicted values for chickens, peafowls, and pigeons (predicted values between 17-27 Hz).

Another line of evidence is from middle ear peak vibration and columella length. Observe that the ostrich has a peak of middle ear vibration at a lower frequency than both the pigeon and chicken (see fig 3 of Zeyl et al 2020) and refs inside. In a study of 38 species of bird middle ears (*11*), columella length was found to be negatively related to the peak frequency of maximum vibration in the middle ear.

The best frequency of middle ear vibration is an important, but not the only, predictor of best sensitivity. Using our measure of wandering albatross columella length (11.6 mm), peak frequency of middle ear vibration is lower in albatrosses (475 Hz) vs pigeons (1241) or chickens (1183 Hz). All else being equal, this could shift the audiogram to lower frequencies in albatrosses.

Table S1 Model output for predicted relationship between frequency at 60 dB and columella volume, taken from Zeyl et al. 2023, Hearing Research.

	Estimate	Std error	t value	p value
Intercept	1.934	0.554	3.491	0.039
Log(CV)	-0.714	0.221	-3.236	0.048

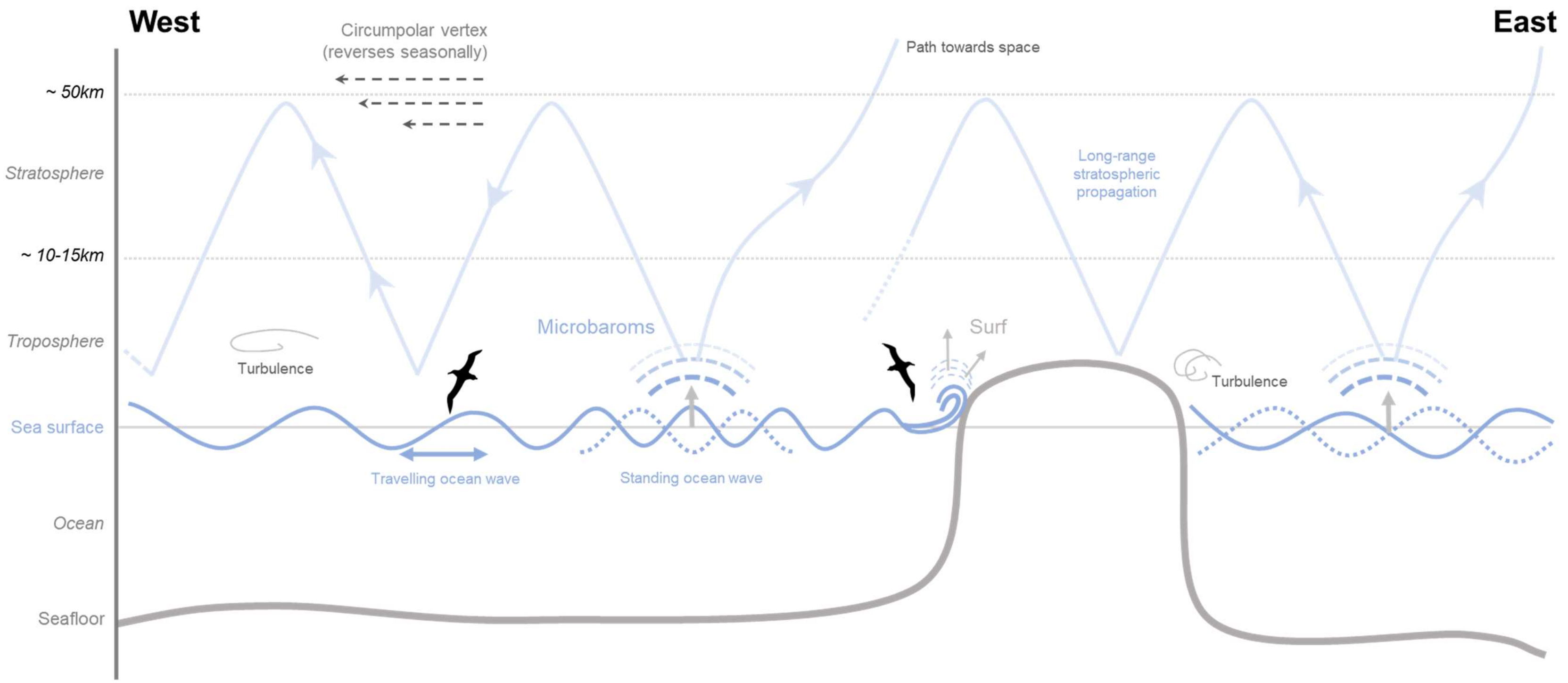

Figure S1. Figure adapted from Patrick et al. (2021), *Front Ecol Evol*, with permission. Schematic to show the marine infrasonic wavefield. Microbaroms and surf (blue) are produced as continuous acoustic background noise. Downwind (i.e. east to west), microbaroms propagate over long distances; in the upwind direction they are not guided and propagate towards space. Wind direction changes seasonally (see Supplementary Text).

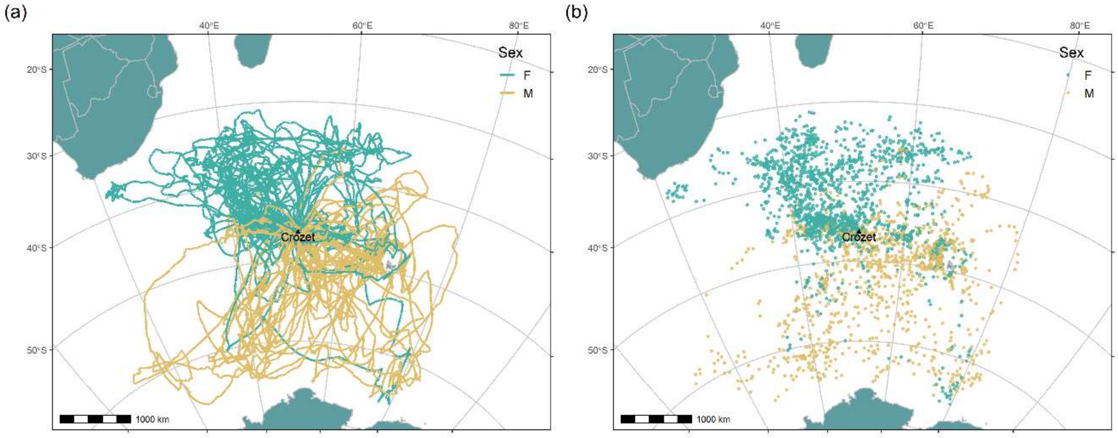

Figure S2. (a) Foraging trips from incubating wandering albatross Diomedea exulans from Crozet Island (black triangle) in 2013. GPS fixes were collected between 2013-01-19 and 2013-03-30. **(b)** Location of decision points used in analysis. Teal tracks/points are females, yellow tracks/points are males. Map displayed in the Azimuthal Equal Area projection, centred on Crozet.

Table S2. Summary metrics for complete foraging trips and travel bouts of >20km, from which decision points were derived. Data are presented for males and females separately. Values presented as mean ± standard deviation.

		**Max distance from colony (km)**	**Total distance (km)**	**Duration (hours)**
**Females**	*Foraging trips*	1082.13±624.25	5569.64±3549.25	238.25±116.20
	Travel bouts	836.34±557.37	110.67±121.79	2.11±2.30
**Males**	*Foraging trips*	1324.76±914.48	6138.45±3807.86	240.92±93.00
	Travel bouts	962.06±731.78	138.84±172.96	2.46±2.93

Sensitivity analysis for segment size. We used a 60° aperture to define segments of the decision point, giving 6 segments for comparison. However, as this segment size was ultimately an arbitrary choice, we conducted a post-hoc sensitivity analysis to determine whether varying the size and therefore number of segments led to variation in the output of our best-supported models. We fitted the best supported models for males and females to data in which the aperture size for segment varied between 20 and 90°.

Our sensitivity analysis suggested that varying the aperture denoting segments did not have a substantial effect on the results of our analysis (Fig S2).

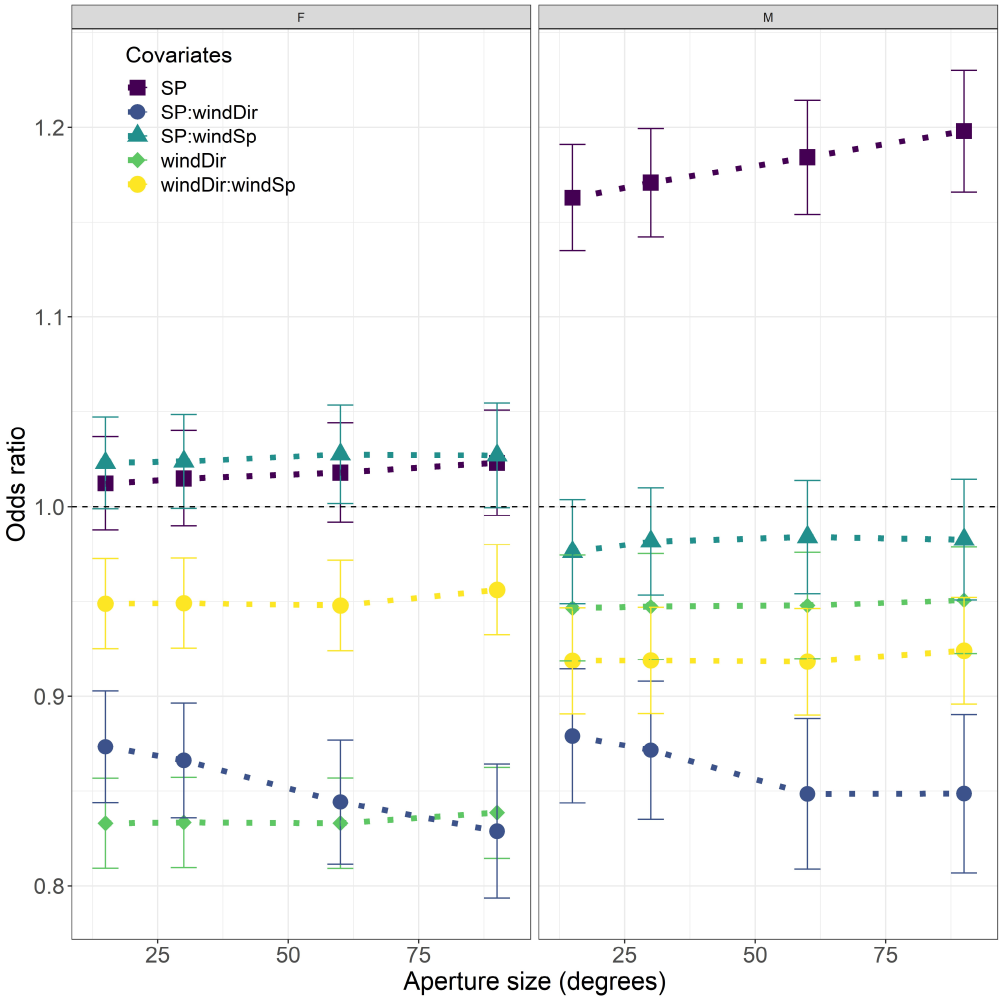

Figure S3. Coefficient estimates (odds ratios) from post-hoc sensitivity analyses. Compares output of best models for females (F, left panels) and males (M, right panels) using datasets where aperture size denoting segments was varied between 15 and 90°. Only aperture sizes into which 360 could be divided evenly could be used to calculate segments.

Controlling for trip stage. If albatrosses use microbarom infrasound as a movement cue, it is possible that its utility differs during middle stages of trips versus outbound and return commutes, when birds move away from or return to the colony. During the middle part of the trip, birds are likely to be foraging or searching for patches and may be more greatly influenced by olfactory and visual cues. We therefore attempted to divide each trip into 3 different states: outbound (moving out of the colony), middle, and inbound (moving towards the colony), following the best available methods for this species (1). However, due to the long looping foraging trips frequently exhibited by wandering albatrosses (Figure S4), this method was not deemed to be appropriate for our species and populations, splitting the trips into stages representing approximately 1/3 of fixes each, which is unlikely to reflect informative differences in behaviour during the trip. Trips were therefore modelled in their entirety, with no splitting of putative trip stages.

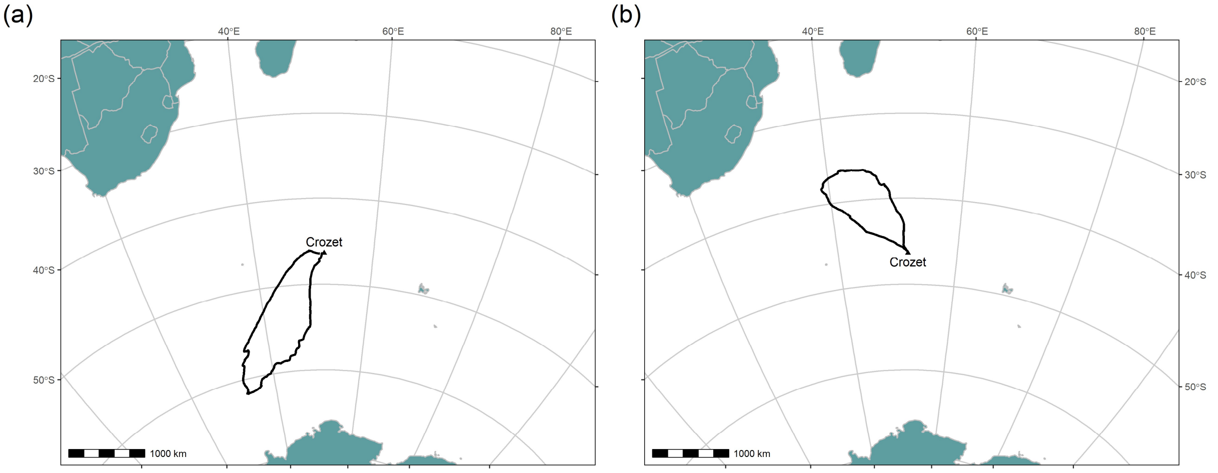

Figure S4. Example trips for two birds to illustrate looping foraging style: (a) male, commencing 2013-02-23 10:41:49 UTC; (b) female, commencing 2013-03-11 08:56:02 UTC. Map displayed in the Azimuthal Equal Area projection, centred on Crozet.

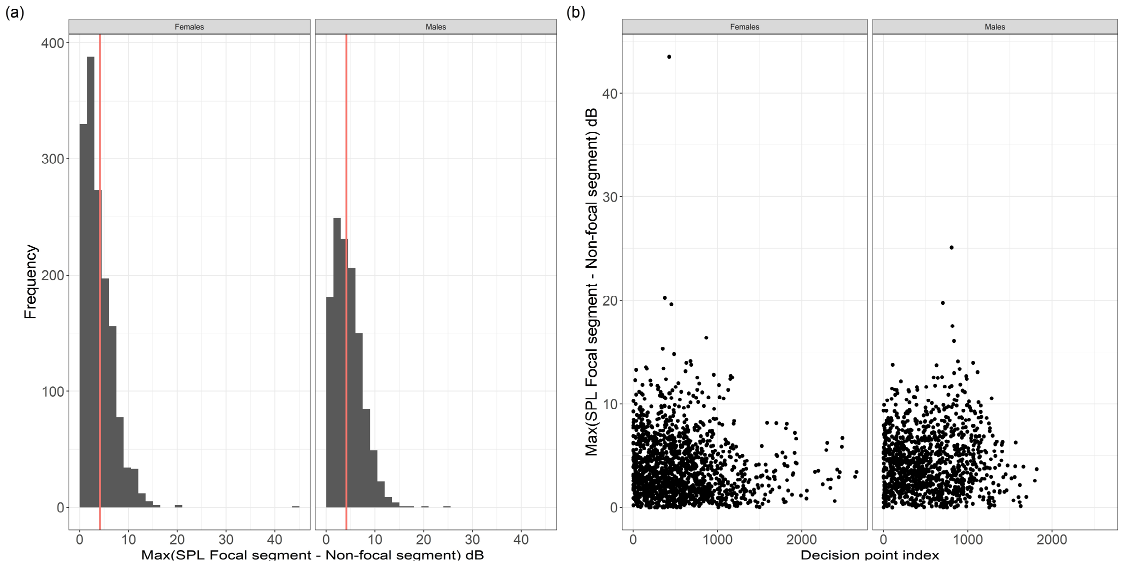

Figure S5. (A) Frequency histogram of maximum SPL differences (dB) measured between focal and non-focal segments. (B) Scatter plot of maximum SPL differences against decision point index. SPL differences calculated in pascals and transformed to dB. Red line in panel (A) shows mean SPL difference for males vs females**.**

Movie S1. Animation to show hourly variation in the microbarom infrasonic soundscape (0.06-1.1 Hz) from the perspective of a foraging albatross. Animation begins 2013-02-01 15:00 UTC and runs to 2013-02-16 10:00 UTC. Leftmost panel shows the initial hourly infrasound microbarom source model integrated between 0.1 and 1Hz, according to Waxler et al., 2007 and implemented by Smets, 2018. Middle panel shows the infrasound propagation loss model by Taillipied et al., 2017, from the perspective of the bird's GPS location, again integrated between 0.1 and 1Hz. Arrows superimposed on the propagation loss model indicate the wind direction and speed within the stratosphere and troposphere. Rightmost panel shows the soundscapes from the bird's perspective, between 0.1 and 1 Hz. The star indicates the GPS position of the bird at the presented timestamp. The dots show the previous positions, showing the track. The triangle indicates Kerguelen islands, where IMS infrasound station I23FR is stationed for the CTBTO. SPL is used for illustration for interpretability, analyses were conducted using sound pressure measured as pascals.

References

1. O. F. C. den Ouden, P. S. M. Smets, J. D. Assink, L. G. Evers, A Bird's-Eye View on Ambient Infrasonic Soundscapes. *Geophys. Res. Lett.* **48** (2021), doi:10.1029/2021GL094555.
2. O. F. C. den Ouden, J. D. Assink, P. S. M. Smets, L. G. Evers, A climatology of microbarom detections at the Kerguelen Islands: unravelling the ambient noise wavefield. *Geophys. J. Int.*, 1–41 (2022).
3. F. B. Jensen, W. A. Kuperman, M. B. Porter, H. Schmidt, *Computational Ocean Acoustics* (Springer New York, New York, NY, 2011; https://link.springer.com/10.1007/978-1-4419-8678-8).
4. A. D. Pierce, *Acoustics* (Springer International Publishing, Cham, 2019; http://link.springer.com/10.1007/978-3-030-11214-1).
5. L. C. Sutherland, H. E. Bass, Atmospheric absorption in the atmosphere up to 160 km. *J. Acoust. Soc. Am.* **115**, 1012–1032 (2004).
6. R. Waxler, J. Assink, in *Infrasound Monitoring for Atmospheric Studies* (Springer International Publishing, Cham, 2019; http://link.springer.com/10.1007/978-3-319-75140-5_15), pp. 509–549.
7. V. E. Ostashev, D. K. Wilson, *Acoustics in Moving Inhomogeneous Media* (CRC Press, 2015; https://www.taylorfrancis.com/books/9781482266658).
8. D. Tailpied, A. Le Pichon, E. Marchetti, J. Assink, S. Vergniolle, Assessing and optimizing the performance of infrasound networks to monitor volcanic eruptions. *Geophys. J. Int.* **208**, 437–448 (2017).
9. O. F. C. Den Ouden, J. D. Assink, P. S. M. Smets, L. G. Evers, A climatology of microbarom detections at the Kerguelen Islands: Unravelling the ambient noise wavefield. *Geophys. J. Int.* **229**, 1646–1664 (2022).
10. R. Waxler, K. Gilbert, C. Talmadge, C. Hetzer, in *8th International Conference on Theoretical and Computational Acoustics (ICTCA), Crete, Greece.* (2007).
11. J. Peacock, G. M. Spellman, D. J. Tollin, N. T. Greene, A comparative study of avian middle ear mechanics. *Hear. Res.* **395**, 108043 (2020).
12. J. N. Zeyl, E. P. Snelling, R. Joo, S. Clusella-Trullas, Scaling of ear morphology across 127 bird species and its implications for hearing performance. *Hear. Res.* **428** (2023), doi:10.1016/j.heares.2022.108679.
