# Supplementary figures and images for: Albatross movement suggests sensitivity to infrasound cues at sea
Source: Proc Natl Acad Sci U S A. 2023 Oct 9;120(42):e2218679120. doi: 10.1073/pnas.2218679120 (PMC10589618; doi:10.1073/pnas.2218679120)

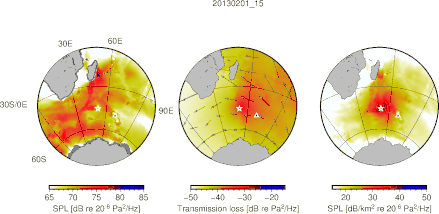

Supplement: Movie S1. — Animation to show hourly variation in the microbarom infrasonic soundscape (0.06-1.1 Hz) from the perspective of a foraging albatross. Animation begins 2013-02-01 15:00 UTC and runs to 2013-02-16 10:00 UTC. Leftmost panel shows the initial hourly infrasound microbarom source model integrated between 0.1 and 1Hz, according to Waxler et al., 2007 and implemented by Smets, 2018. Middle panel shows the infrasound propagation loss model by Taillipied et al., 2017, from the perspective of the bird’s GPS location, again integrated between 0.1 and 1Hz. Arrows superimposed on the propagation loss model indicate the wind direction and speed within the stratosphere and troposphere. Rightmost panel shows the soundscapes from the bird’s perspective, between 0.1 and 1 Hz. The star indicates the GPS position of the bird at the presented timestamp. The dots show the previous positions, showing the track. The triangle indicates Kerguelen islands, where IMS infrasound station I23FR is stationed for the CTBTO. SPL is used for illustration for interpretability, analyses were conducted using sound pressure measured as pascals. [file pnas.2218679120.sm01.gif]
